# A Robust Method for the Unsupervised Scoring of Immunohistochemical Staining

**DOI:** 10.3390/e26020165

**Published:** 2024-02-15

**Authors:** Iván Durán-Díaz, Auxiliadora Sarmiento, Irene Fondón, Clément Bodineau, Mercedes Tomé, Raúl V. Durán

**Affiliations:** 1 Signal Theory and Communications Department, University of Seville, Avda. Descubrimientos S/N, 41092 Seville, Spain; sarmiento@us.es (A.S.); irenef@us.es (I.F.); 2 Department of Pathology, Brigham and Women’s Hospital, Boston, MA 02115, USA; cbodineau@bwh.harvard.edu; 3 Department of Genetics, Harvard Medical School, Boston, MA 02115, USA; 4 Centro Andaluz de Biología Molecular y Medicina Regenerativa—CABIMER, Consejo Superior de Investigaciones Científicas, Universidad de Sevilla, Universidad Pablo de Olavide, 41092 Seville, Spain; mercedes.tome@cabimer.es (M.T.); raul.duran@cabimer.es (R.V.D.)

**Keywords:** histopathological images, principal component analysis, unsupervised stain separation, semi-quantitative scoring

## Abstract

Immunohistochemistry is a powerful technique that is widely used in biomedical research and clinics; it allows one to determine the expression levels of some proteins of interest in tissue samples using color intensity due to the expression of biomarkers with specific antibodies. As such, immunohistochemical images are complex and their features are difficult to quantify. Recently, we proposed a novel method, including a first separation stage based on non-negative matrix factorization (NMF), that achieved good results. However, this method was highly dependent on the parameters that control sparseness and non-negativity, as well as on algorithm initialization. Furthermore, the previously proposed method required a reference image as a starting point for the NMF algorithm. In the present work, we propose a new, simpler and more robust method for the automated, unsupervised scoring of immunohistochemical images based on bright field. Our work is focused on images from tumor tissues marked with blue (nuclei) and brown (protein of interest) stains. The new proposed method represents a simpler approach that, on the one hand, avoids the use of NMF in the separation stage and, on the other hand, circumvents the need for a control image. This new approach determines the subspace spanned by the two colors of interest using principal component analysis (PCA) with dimension reduction. This subspace is a two-dimensional space, allowing for color vector determination by considering the point density peaks. A new scoring stage is also developed in our method that, again, avoids reference images, making the procedure more robust and less dependent on parameters. Semi-quantitative image scoring experiments using five categories exhibit promising and consistent results when compared to manual scoring carried out by experts.

## 1. Introduction

The detection of biomarker expression in tissue images is commonly used in both research laboratories and in the clinic. This detection is carried out through the use of different color chromogens that bind to the antigens of interest using an antibody–antigen detection system, a technique known as immunohistochemistry (IHC) [1]. This allows one to determine the presence of the chromogen and the protein to which it binds by observing the tissue using bright field microscopy. Capturing tissue images allows for the preservation and analysis of the expression patterns of the analyzed proteins. Usually, the presence, concentration and distribution of the chromogen are determined manually by experts by viewing and comparing images.

**Figure 1:**
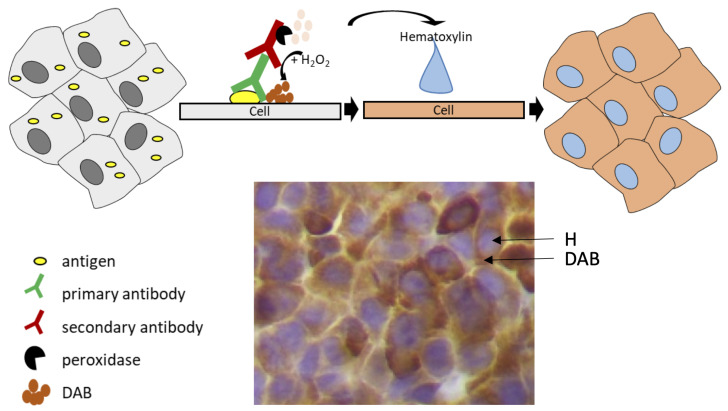
Schematic representation of immunohistochemistry: the antigen is specifically recognised by the primary antibody. Following secondary antibody binding, peroxidase activity leads to DAB precipitation (brown) next to the antigen. Subsequently, the tissue is stained with the hematoxylin dye to detect cell nuclei (blue-purple).

Two widely used chromogens are 3,3′-Diaminobenzidine (DAB) and Hematoxylin (H) [2,3,4,5,6]. The presence of DAB is detected as a brown stain, while H appears as blue. When combined, H staining marks cell nuclei, whereas DAB staining reveals the presence and distribution of the protein of interest. Figure 1 illustrates the staining procedure.

The IHC image evaluation and quantification procedure lacks objectivity, as it is a subjective method performed by experts and is subject to observer variations. Indeed, even the same observer can evaluate the same image differently at different times [7]. Furthermore, manual scoring is affected by visual distortion due to the perception of colors and their intensity depending on surrounding colors [8]. This is particularly significant in protocols that use two dyes. Finally, IHC visual scoring is a time-consuming procedure for researchers. For this reason, the demand for automated scoring procedures is increasing in laboratories and in the clinic [9]. This type of algorithm must include a stage to separate both stains to perform the scoring with a greater reliability.

Scoring systems can be classified as those based on the perceived staining intensity and those based on stained cell percentages, while some others are a mix of both. Regarding the most used categories, they range from the simplest systems that class tissue samples as positive or negative, depending on whether or not the observed staining exceeds a certain threshold, to those that use a set of scoring levels depending on the staining amount—so-called semi-quantitative scoring systems [8]. Although some platforms and applications for the semiquantitative scoring of IHC images already exist, they require reference images as well as the intervention of researchers. Examples of these systems are QuPath [10], IHC Profiler [11] and DAB-quant [12].

The staining separation stage is necessary in automated scoring systems since it allows for the obtaining of image features based on each stain. This separation stage cannot be performed in the RGB color space because the concentration of the light-absorbing material follows the Beer–Lambert law (although not exactly, due to some degree of dispersion). For this reason, a transform based on this law is applied to the RGB image, obtaining the coordinates of each pixel in a new space, i.e., the optical density (OD). Within this space, the intensity of each coordinate can be considered as a linear combination of the intensities due to each stain [13].

Separation methods in the literature can be classified into two categories: supervised and unsupervised (or blind) methods [14,15]. In the first case, the coordinates of each used stain are known in the OD space; otherwise, they require the intervention of an observer or the use of reference images to calculate these coordinates. In the second case, unsupervised methods use techniques like independent component analysis (ICA) [16,17], non-negative matrix factorization (NMF) [13,18] or non-negative least squares (NNLS). These techniques suffer from a strong dependence on initialization. Thus, these methods require some prior knowledge about the stain vector coordinates to perform a correct separation, i.e., to guide the search towards the correct solution. Some approaches based on deep learning [4,19] require a large number of training images, so they cannot be considered unsupervised techniques.

In the context of supervised methods, some approaches have exploited singular value decomposition (SVD) in order to calculate the subspace where the OD data lie [20,21]. Principal component analysis (PCA) works in the same way as SVD, searching for the principal directions of the data, i.e., those that preserve the greatest quantity of variance or power (when uncentered PCA is performed) [22].

In this work, we propose an unsupervised method for comparative biomarker quantification that is an improvement over the method presented in our previous work [14]. Accordingly, this new algorithm represents a simpler and more robust method for color separation, with a very low dependence on the selected parameters, and more accurate quantification without the need for reference or control images.

We circumvent the use of NMF in the staining separation stage, the results of which are very dependent on the starting point. Instead, the proposed method exploits DAB (brown) and H (blue) concentrations to estimate the vectors associated with the color of both stains. This stage is highly robust in order to achieve consistent stain vector estimates with no prior knowledge, even avoiding the use of reference images that are required for the method in [14].

For the scoring stage, using features whose values mostly increase with the stain concentration helped us to develop a procedure for the initialization of the clustering algorithm (k-means). While, in our previous work, a set of three parameters, together with the use of two reference images, were needed, in the present work, the initialization procedure provides excellent results for common values of the two parameters that we use and, again, avoids the use of reference images. This makes any intervention of the observer totally unnecessary and allows for a totally automated procedure.

## 2. Methods

**Figure 2:**
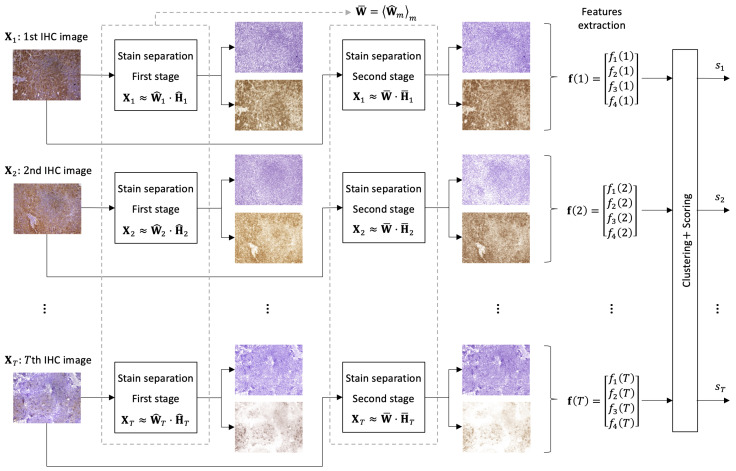
Scheme of the procedure described in this work. The color separation step allows each image to be described in terms of the superposition of H and DAB stains. This step consists of two stages. In the first stage, the data matrix of each image is decomposed separately (the color matrix, ${\hat{W}}_{m}$, is estimated for each image by exclusively using this image). The estimated color matrices are averaged by $\bar{W}={\langle {\hat{W}}_{m}\rangle }_{m}$. In the second color separation stage, the data matrix of each image is decomposed using the average color matrices. From the results obtained after color separation, the feature vector of each image is extracted to perform clustering and scoring.

We developed an automated and unsupervised procedure for scoring IHC images with two stains: hematoxylin and DAB. Image set quantification is performed in three steps: stain separation, feature extraction and, finally, clustering and scoring. A semi-quantitative scoring system clusters the images in five groups and assigns scores from “1+” to “5+” for each image, with “1+” being the lowest level of DAB expression. Figure 2 shows the scheme of the proposed method.

### 2.1. Stain Separation

The stain separation step is carried out within the color space given by the transform provided by the Beer–Lambert Law. Let $I_{m}\in R^{3\times N}$ be the matrix of RGB intensities of the *m*th analyzed image, where *N* is the total number of pixels of each color plane. Then, the relative optical density matrix of this image, $X_{m}$, is obtained as

(1) $$X_{m}=-\log\left(\frac{I_{m}}{I_{o}}\right),$$

where $I_{o}$ is the maximum level of intensity at each pixel (255 for 8-bit images) and the function $\log(I_{m})$ is applied to each entry of the matrix $I_{m}$. This provides a representation of each pixel in a $R^{3}$ space, where each color corresponds to a direction, with a higher vector norm representing a darker color. Thus, each color (including its darkness) can be represented by the coordinates of its corresponding vector within this space. Since the image was obtained via an IHC procedure, the color vector of each pixel lies, mainly, within the subspace spanned by the color vectors associated with both stains, i.e., the subspace due to H (blue) and DAB (brown). This allows us to estimate the matrix $X_{m}$ as [13]

(2) $${\hat{X}}_{m}=W_{m}H_{m},$$

where ${\hat{X}}_{m}$ is the matrix that estimates $X_{m}$, $W_{m}$ is a 3×2 matrix whose columns are the color vectors associated with both stains for the *m*th image and $H_{m}$ is a 2×N matrix whose rows are the intensities or activations of each column of $W_{m}$ at each pixel of the *m*th image.

**Figure 3:**
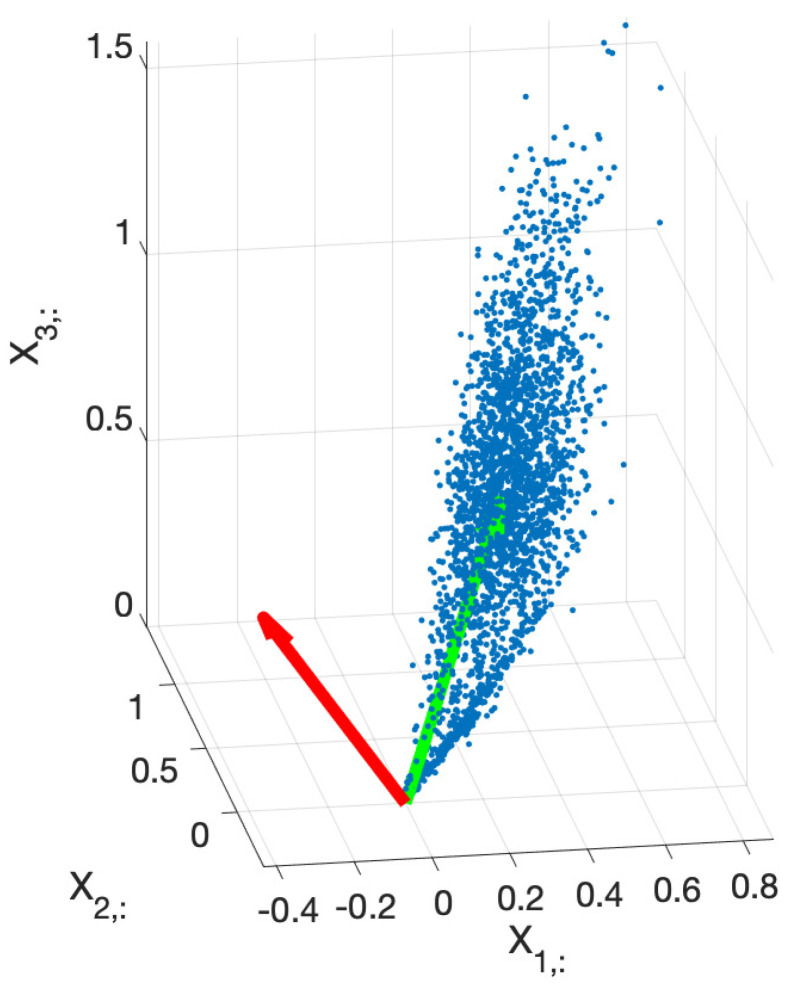
Scatter plot (blue) of the columns of matrix $X_{m}$ for one example image with DAB and H stains. For better visualization, the entries were sampled prior to building the scatter plot. As can be seen, the points lie mainly in the plane defined by the stain vectors. The principal directions (green and red) along the two first principal components are depicted. These orthogonal directions lie in the same plane defined by the color stain vectors, although they do not coincide. The principal components allow us to simplify the problem of searching the stain vectors to a 2D problem.

We exploit this in order to simplify the problem by means of dimension reduction. The subspace generated by the columns of $W_{m}$ is found by applying uncentered principal component analysis (PCA) to the matrix $X_{m}$. This provides the principal directions of the data that lie within the above-mentioned subspace (although the principal directions do not have to coincide with the columns of $W_{m}$), as can be seen in Figure 3.

Let

(3) $$Q_{m}=\left[\begin{array}{ccc} {q_{m}}_{1} & {q_{m}}_{2} & {q_{m}}_{3} \end{array}\right]$$

be the unitary matrix whose columns are the eigenvectors of $R_{m}=X_{m}X_{m}^{T}/N$ and

(4) $$L_{m}=\mathrm{diag}\left\{{L_{m}}_{1},{L_{m}}_{2},{L_{m}}_{3}\right\}$$

be the diagonal matrix whose non-zero entries are the eigenvalues of $R_{m}$. The superscript *T* stands for transpose. Both the eigenvectors and the eigenvalues are in descending order. Since $X_{m}\approx {\hat{X}}_{m}$, the principal subspace is given by the two first columns of $Q_{m}$, as the third eigenvalue, ${L_{m}}_{3}$, is very small compared to ${L_{m}}_{1}$ and ${L_{m}}_{2}$. The columns of $X_{m}$ are projected onto the principal subspace (the plane defined by the first two principal directions), and the 2×N matrix of principal components

(5) $$Z_{m}=\left[\begin{array}{c} \sqrt{{{L_{m}}_{1}}^{-1}}\cdot {q_{m}^{T}}_{1}\\ \sqrt{{{L_{m}}_{2}}^{-1}}\cdot {q_{m}^{T}}_{2} \end{array}\right]\cdot X_{m}.$$

is thus obtained.

**Figure 4:**
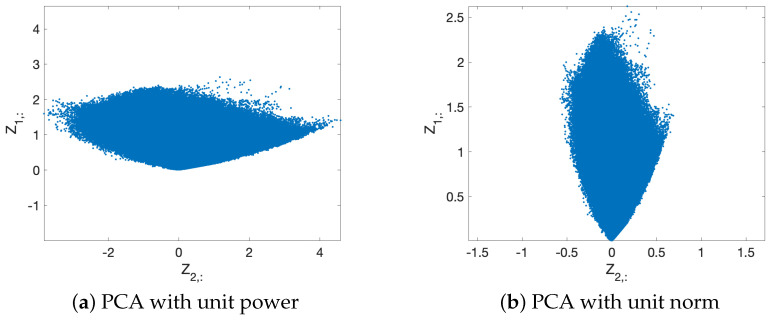
Scatter plot of the principal components for an example image when (**a**) PCA with unit power principal components and (**b**) PCA with unit norm loading vectors are performed. The normalization of the principal component powers allows us to spread the scattering angle so the angle between the stain vectors is greater, improving the accuracy of the estimate. On the other hand, the normalization of the norm of the loading vectors maintains the distance between the stain vectors, which reduces the accuracy. The second principal component is depicted along the horizontal axis for visual convenience.

Since all columns of $X_{m}$ lie in the first octant of $R^{3}$ Euclidean space, both color vectors are relatively close. A simple projection onto the $R^{2}$ subspace defined by ${q_{m}}_{1}$ and ${q_{m}}_{2}$, maintaining the scales of the principal components, would lead to a 2D scattering where it would be difficult to precisely distinguish the color vectors associated with each stain. This is the reason why the columns of $X_{m}$ are projected onto the subspace defined by ${q_{m}}_{1}\sqrt{{L_{m}}_{1}}$ and ${q_{m}}_{2}\sqrt{{L_{m}}_{2}}$, i.e., PCA with unit-power principal components is performed [22]. This expands the scattering in the second principal component direction, as we show in Figure 4, and allows us to recover the stain color vectors more accurately.

#### 2.1.1. Stain Color Basis Estimation

The next step of the procedure consists of estimating the two basis vectors for the stain colors, i.e., the columns of the matrix $W_{m}$. Due to the projection in (5), the columns of this matrix are projected onto the same subspace defined by the principal directions

(6) $$B_{m}=\left[\begin{array}{c} \sqrt{{{L_{m}}_{1}}^{-1}}\cdot {q_{m}^{T}}_{1}\\ \sqrt{{{L_{m}}_{2}}^{-1}}\cdot {q_{m}^{T}}_{2} \end{array}\right]\cdot W_{m}.$$

**Figure 5:**
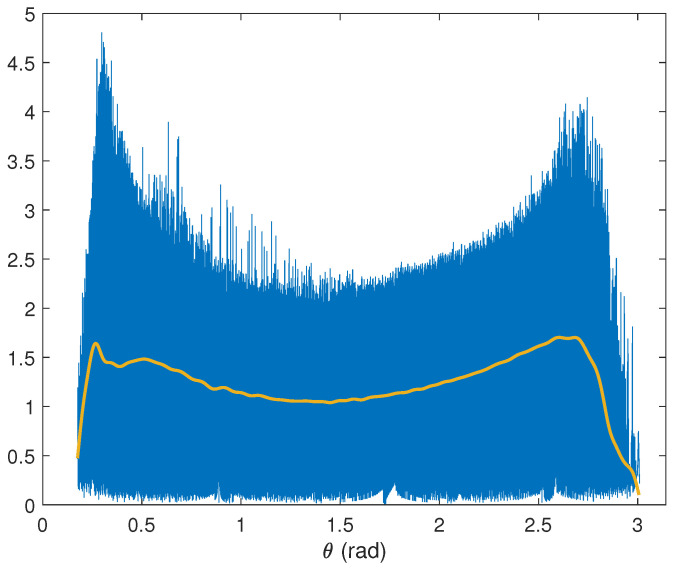
Norm $r_{m}\left(n\right)$ (blue) of the columns of $Z_{m}$ for an example image versus the angle of the columns together with the smoothed mean of concentration, $v_{m}\left(k\right)$ (yellow). Searching for the peaks of this last function leads to the angles corresponding to the stain vectors within the subspace of principal components.

Let $\theta_{m}\left(n\right)$ and $r_{m}\left(n\right)$ be the angle and norm of the *n*th column of $Z_{m}$. Since, in many pixels, only one stain is dominant, the values of $r_{m}\left(n\right)$ are higher around the basis vectors. Thus, the columns of $B_{m}$ are estimated using the angles where high concentrations of $r_{m}\left(n\right)$ are found. The range of values of $\theta_{m}\left(n\right)$ is divided into 1000 bins to achieve an appropriate angle resolution. For the *k*th bin, whose center is given by $\phi_{m}\left(k\right)$, the mean of the values of $r_{m}\left(n\right)$, such that $\theta_{m}\left(n\right)$ lies within the bin, is computed. Then, this function is smoothed by a lowpass eighth-order Butterworth filter with a digital cutoff frequency equal to 0.035 to obtain the function $v_{m}\left(k\right)$, as illustrated in Figure 5.

Therefore, $B_{m}$ is estimated as

(7) $${\hat{B}}_{m}=\left[\begin{array}{cc} \cos({\phi_{m}}_{1}) & \cos({\phi_{m}}_{2})\\ \sin({\phi_{m}}_{1}) & \sin({\phi_{m}}_{2}) \end{array}\right]$$

where ${\phi_{m}}_{1}$ and ${\phi_{m}}_{2}$ are the main peaks in the filtered version of $r_{m}\left(n\right)$. Since the existence of more than two peaks is possible, in order to avoid a bad selection of the true angles of both basis vectors, a procedure was implemented. Due to the nature of the data, the basis vectors are always near the extrema of $\phi_{m}\left(n\right)$ (in an ideal case, the points of the scattering are a linear combination of the basis vectors with non-negative coefficients). Thus, the maximum of $v_{m}\left(k\right)$ is selected as the position for one of the basis vectors. The other peak is selected from the angle bins that lie within the opposite half of the total range of $\phi_{m}\left(k\right)$. This ensures that the selected angles are a good estimate of the basis vectors.

It is easy to see that the estimate of $W_{m}$, related to ${\hat{B}}_{m}$, is given by the representation of the columns of this matrix in the whole 3D space as

(8) $${\hat{W}}_{m}=\left[\begin{array}{cc} {q_{m}}_{1}\sqrt{{L_{m}}_{1}} & {q_{m}}_{2}\sqrt{{L_{m}}_{2}} \end{array}\right]\cdot {\hat{B}}_{m}.$$

#### 2.1.2. Color Deconvolution

From (2), we can write the decomposition of $X_{m}$ in terms of ${\hat{W}}_{m}$ and $H_{m}$ as

(9) $$X_{m}={\hat{W}}_{m}\cdot H_{m}.$$

This describes, for each column of $H_{m}$, an overdetermined system of equations, as $W_{m}$ and $X_{m}$ are known. Therefore, the stain concentration matrix related to the *m*th image is estimated by means of a linear least squares problem as

(10) $${\hat{H}}_{m}={\hat{W}}_{m}^{+}X_{m}$$

where ${\hat{W}}_{m}^{+}={\left({\hat{W}}_{m}^{T}{\hat{W}}_{m}\right)}^{-1}\cdot {\hat{W}}_{m}$ is the Moore–Penrose pseudoinverse of the full-rank matrix ${\hat{W}}_{m}$ [23]. This procedure is known as color deconvolution [24]. Since the concentration of each stain at every pixel must be non-negative, the values of ${\left[{\hat{H}}_{m}\right]}_{ij}<0$ are set to 0.

The estimate is improved by means of the following procedure. The values of ${\left[{\hat{H}}_{m}\right]}_{ij}$ for columns of ${\hat{H}}_{m}$ that only have one non-zero entry are recalculated by using the corresponding column of ${\hat{W}}_{m}$. After this, a new estimate of the basis vectors is computed as the mean of the columns of $Z_{m}$ for which one stain is dominant, i.e., when the value of a concentration is, at least, 10 times that of the other. A new estimate of ${\hat{H}}_{m}$ is computed following the procedure described in Equation (10).

#### 2.1.3. Average Basis Vectors

Once the previous procedure is carried out, the average of the ${\hat{W}}_{m}$ matrices is computed

(11) $$\bar{W}={\langle {\hat{W}}_{m}\rangle }_{m}.$$

Then, a new concentration matrix is estimated for each image as ${\bar{H}}_{m}={\bar{W}}^{+}X_{m}$. Again, the values of ${\left[{\bar{H}}_{m}\right]}_{ij}<0$ are set to 0 and then the values of ${\left[{\bar{H}}_{m}\right]}_{ij}$ for the columns of ${\bar{H}}_{m}$ that only have one non-zero entry are recalculated.

### 2.2. Feature Extraction

A set of four features is computed for each image. Since the columns of ${\hat{W}}_{m}$ and $\bar{W}$ have not been normalized, we take them into account together with ${\hat{H}}_{m}$ and ${\bar{H}}_{m}$. The *j*th column of ${\hat{W}}_{m}$ (resp. $\bar{W}$) is denoted as ${\hat{W}}_{m_{:,j}}$ (resp. ${\bar{W}}_{:,j}$) and the *i*th row of ${\hat{H}}_{m}$ (resp. ${\bar{H}}_{m}$) is denoted as ${\hat{H}}_{m_{i,:}}$ (resp. ${\bar{H}}_{m_{i,:}}$). Let $M_{1}\left(m\right)$ be the mean of the average intensity of all the pixels of the DAB stain for the *m*th image according to the decomposition using the average basis vectors

(12) $$M_{1}\left(m\right)=\frac{1}{3N}{∥{\bar{W}}_{:,2}\cdot {\bar{H}}_{m_{2,:}}∥}_{1}=\frac{1}{3N}{∥{\bar{W}}_{:,2}∥}_{1}\cdot {∥{\bar{H}}_{m_{2,:}}∥}_{1}=\frac{1}{3N}\sum_{i=1}^{3}{\left[\bar{W}\right]}_{i2}\sum_{j=1}^{N}{\left[{\bar{H}}_{m}\right]}_{2j}$$

where the 1-norm of the matrix is computed entry-wise, i.e., it is the sum of the entries of the matrix (they are all non-negative). For row vectors, we computed the norm the same way as for column vectors. Then, the first feature of the *m*th image is defined as the average of the DAB intensity in this image, normalized by the maximum value that this quantity reaches among the entire set of images

(13) $$f_{1}\left(m\right)=\frac{M_{1}\left(m\right)}{\max_{m}\left\{M_{1}\left(m\right)\right\}}.$$

The second feature is based on the relation between the robust maxima (99th percentile) of both stains (H and DAB). Let $R_{1}\left(m\right)$ (resp. $R_{2}\left(m\right)$) be the robust maximum of the intensity matrix for H staining (resp. DAB staining) according to the results for the average basis vectors, i.e., the robust maximum of the matrix ${\bar{W}}_{:,1}\cdot {\bar{H}}_{m_{1,:}}$ (resp. ${\bar{W}}_{:,2}\cdot {\bar{H}}_{m_{2,:}}$). Then, we define

(14) $${\bar{R}}_{2}\left(m\right)=\frac{R_{2}\left(m\right)}{\max_{m}R_{2}\left(m\right)}$$

and

(15) $${\bar{R}}_{1}\left(m\right)=\frac{R_{1}\left(m\right)}{R_{1}\left(m=\arg\max_{m}R_{2}\left(m\right)\right)}.$$

The second feature is then defined as

(16) $$f_{2}\left(m\right)=\frac{{\bar{R}}_{2}\left(m\right)}{{\bar{R}}_{1}\left(m\right)}.$$

The third feature is analogue to the first one, but using the 2-norm, i.e., based on the average power of the intensity. Let $M_{2}\left(m\right)$ be

(17) $$\begin{array}{cc} M_{3}\left(m\right)=\frac{1}{\sqrt{3N}}{∥{\bar{W}}_{:,2}\cdot {\bar{H}}_{m_{2,:}}∥}_{F}=\frac{1}{\sqrt{3N}}{∥{\bar{W}}_{:,2}∥}_{2}\cdot & {∥{\bar{H}}_{m_{2,:}}∥}_{2}\\ & =\frac{1}{\sqrt{3N}}\sqrt{\sum_{i=1}^{3}{\left[\bar{W}\right]}_{i2}^{2}}\sqrt{\sum_{j=1}^{N}{\left[{\bar{H}}_{m}\right]}_{2j}^{2}} \end{array}$$

where ${∥\cdot ∥}_{F}$ is the Frobenius norm of the matrix. Then, we define the third feature, $f_{3}\left(m\right)$, as

(18) $$f_{3}\left(m\right)=\frac{M_{3}\left(m\right)}{\max_{m}\left\{M_{3}\left(m\right)\right\}}.$$

Although very good results are achieved when using the above-defined features, the inclusion of a fourth feature improves the clustering procedure. The feature $f_{4}\left(m\right)$ is defined as the square root of the average power of the intensity of the DAB stain at every pixel according to the results of the basis vectors estimated for each image,

(19) $$f_{4}\left(m\right)=\frac{1}{\sqrt{3N}}{∥{\hat{W}}_{m_{:,2}}\cdot {\hat{H}}_{m_{2,:}}∥}_{F}=\frac{1}{\sqrt{3N}}{∥{\hat{W}}_{m_{:,2}}∥}_{2}\cdot {∥{\hat{H}}_{m_{2,:}}∥}_{2}$$

### 2.3. Clustering and Scoring

In general, the values of the four defined features increase when the concentration of the DAB stain increases. Consequently, a simple, automated and efficient procedure was used to initialize the k-means algorithm for clustering. In addition, this allows us to assign the scores in an increasing order of the centroid norms. Let f(m) be the vector of features whose *i*th entry is $f_{i}\left(m\right)$. In order to initialize the clustering algorithm, we computed the *p*-norm of the vector

(20) $${∥f(m)∥}_{p}={\left(\sum_{i=1}^{T}{\left|f_{i}\left(m\right)\right|}^{p}\right)}^{1/p}$$

where *T* is the length of the vector of features f(m). A certain range of values can lead to good initialization and good results for the clustering algorithm. In Section 3, we discuss these values.

When the values of ${∥f(m)∥}_{p}$ are sorted in ascending order, the result is not a linear function of *m*, but rather a power function. For an automated initialization of the clustering algorithm, the range of values of ${∥f(m)∥}_{p}^{q}$ is divided into five intervals with the same length, which provides a good starting point. Therefore, the *k*th interval is $I_{k}=\left[E_{k}^{\left(i\right)},\;E_{k}^{\left(s\right)}\right]$, where

(21) $$\begin{array}{ccc} E_{k}^{\left(i\right)} & = & \frac{k-1}{5}\left(\max{∥f(m)∥}_{p}^{q}-\min{∥f(m)∥}_{p}^{q}\right)+\min{∥f(m)∥}_{p}^{q} \end{array}$$

(22) $$\begin{array}{ccc} E_{k}^{\left(s\right)} & = & \frac{k}{5}\left(\max{∥f(m)∥}_{p}^{q}-\min{∥f(m)∥}_{p}^{q}\right)+\min{∥f(m)∥}_{p}^{q}. \end{array}$$

Although the value of *q* may change for different feature sets, the square root is a good selection for the four proposed features. We fitted the first and second half of the sorted values of ${∥f(m)∥}_{p}$ to a power function and set *q* as the inverse of the average of both resulting powers. For the four selected features, this value is close to 1/2. In Section 3, we discuss the possible values of this parameter. Initial clusters are assigned based on the norm of the feature vector; if this norm lies in the *k*th interval, then the corresponding vector belongs to the *k*th cluster. Thus, the initial *k*th cluster, $\Omega_{k}^{\left(0\right)}$, is set as

(23) $$\Omega_{k}^{\left(0\right)}=\left\{m:\;{∥f(m)∥}_{p}^{q}\in I_{k}\right\}\;\;\;\forall k=1,\ldots ,5.$$

The initial *k*th centroid is set as the mean of the feature vectors that belong to the *k*th cluster

(24) $$c_{k}=\sum_{m\in \Omega_{k}}f(m)\;\;\;\forall k=1,\ldots ,5.$$

These initial centroids were used in the k-means algorithm equipped with a distance measure based on the *p*-norm. Only three iterations of the algorithm are needed to obtain the image scores.

## 3. Results and Discussion

Our dataset was the same as that in [14] and consisted of 94 images taken from stained xenograft tumors. These tumors were generated by subcutaneous implantation of HCT116 cells in mice subjected to either a vehicle or a cell-permeable α-ketoglutarate derivative, dimethylα-KG (DMKG), followed by treatment with the mTORC1 inhibitor temsirolimus (TEM) or with metformin (MET). Tumor samples were then processed for immunohistochemistry as described in [25]. All procedures were approved by the corresponding institutional organizations (APAFIS# 10090 2017052409402562 v2). The omission of the primary antibody in the immunostaining procedure was used as a negative control. Images were acquired in TIFF format with a Leica DM6000B microscope using ×20 or ×40 objective lenses and a Leica DFC500 digital camera. All images were independently scored from “1+” to “5+” by four expert observers. The agreement among observers, measured as the percentage of images that were annotated with the same score by all experts, is 70.21%. This means that the maximum mean agreement of scoring with all observers is 90.96%.

### 3.1. Results of the Stain Separation Step

**Figure 6:**
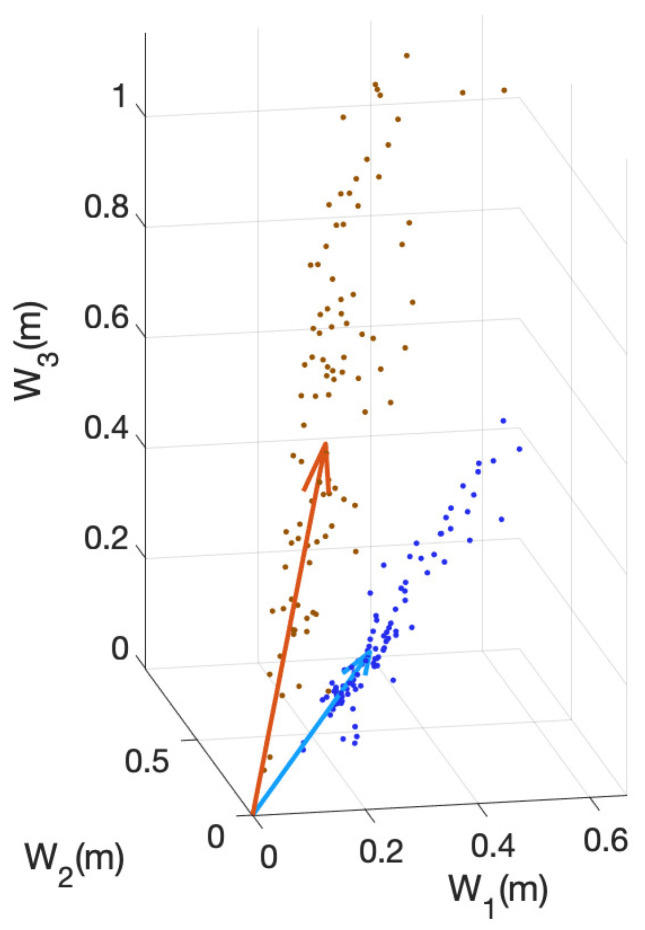
Scatter plot of the estimates ${\hat{W}}_{m}$ for the whole set of analyzed images and for both H (blue) and DAB (brown) stains, together with the columns of the mean $\bar{W}$ for both stains. The consistency of the results for nearly all images can be noted.

**Figure 7:**
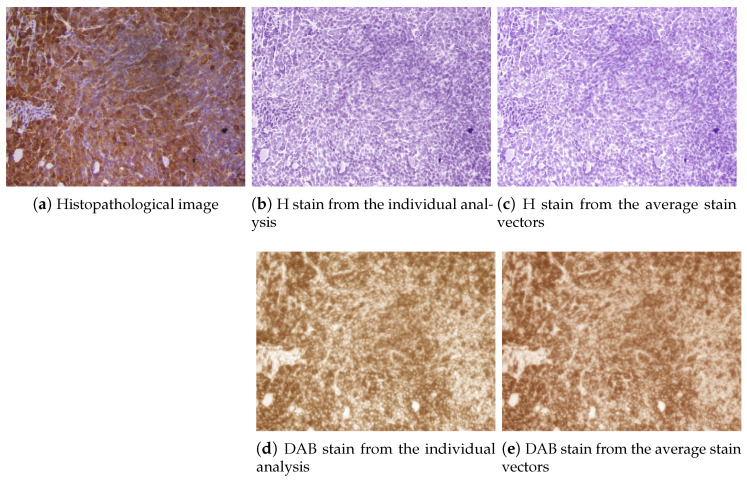
Example of stain separation of an IHC image from the image set: (**a**) original image, (**b**) H stain from the exclusive analysis of this image, (**c**) H stain from the average stain vectors computed in (11), (**d**) DAB stain from the analysis of this image and (**e**) DAB stain from the average stain vectors. This is an example where the analysis of the image essentially coincides with the average, since the intensities of both stains are high.

**Figure 8:**
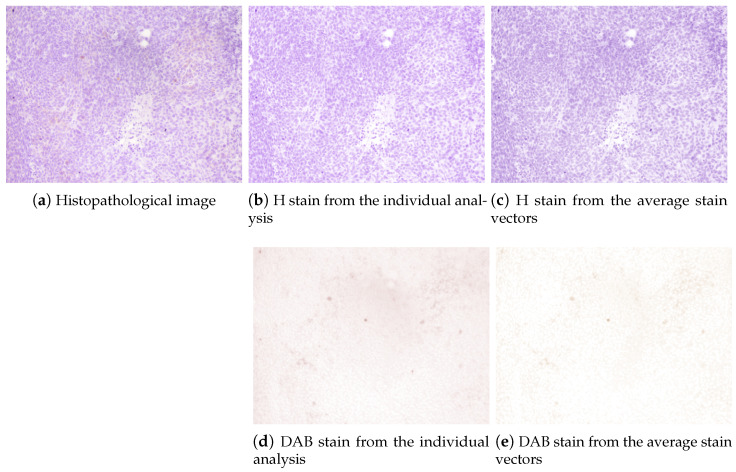
Example of stain separation of another IHC image from the image set: (**a**) original image, (**b**) H stain from the exclusive analysis of this image, (**c**) H stain from the average stain vectors computed in (11), (**d**) DAB stain from the analysis of this image and (**e**) DAB stain from the average stain vectors. This is an example where the exclusive analysis of the image does not provide a good estimate of the DAB stain vector due to the very low concentration of this stain, although the intensity of the separated DAB stain is very similar to that obtained for the average DAB vector.

The stain separation procedure achieved satisfactory results, providing a good decomposition of the DAB and H planes and showing consistent solutions along the whole set of images. In Figure 6, the estimated color stain vectors for each image, together with the mean of these color vectors, are depicted. Note that the direction of the vectors is very similar for all images. In Figure 7 and Figure 8, the results of the separation procedure are shown for two example images from the set. In the first case, the processed image has a high concentration of both (DAB and H) stains, whereas the second image has a very low concentration of the DAB stain. In both figures, two results are shown: the results of an individual analysis without considering the rest of the images based on an estimate from (8), and the estimation of stain concentrations from the average stain vectors estimated in (11). In all cases, the color of the H stain is correctly estimated. Furthermore, even if the color of the DAB stain is not very accurate in the second image after the first analysis, the calculated concentrations of this stain are similar to those obtained from the average stain vectors.

### 3.2. Performance of the Scores Prediction

**Figure 9:**
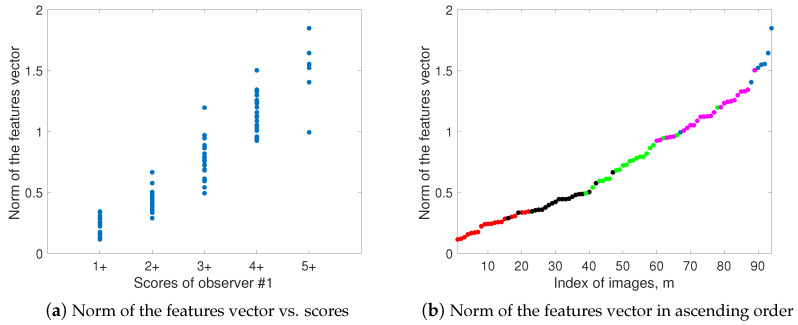
Correlation between the norm of the feature vector and the scores of observer #1 shown as a plot of (**a**) the norm versus the scores and (**b**) the sorted norm versus the image indices. In this last figure, the scores are shown in different colors in ascending order. In both cases, a high correlation between the norm and the scores can be observed. This is used for the automated initialization of the clustering algorithm.

In [14], we showed that the 1-norm of the DAB concentration stain is more correlated than the average threshold method (ATM) score and the pixel-wise H-score with the observers’ scores. There, we used two other features (related to the feature denoted as $f_{3}\left(m\right)$ in the present work, but not the same) that improved the correlation. Here, we show how the correlation of the 1-norm increases when we add the features $f_{2}\left(m\right)$, $f_{3}\left(m\right)$ and $f_{4}\left(m\right)$. All the features that we propose increase (in general) when the score increases, which allows us to use the norm of the feature vector for the automated initialization of the clustering algorithm due to the high correlation between this norm and the score of each image, as can be seen in Figure 9.

Although the procedure was designed to use four features, we also tested its behavior when only two or three features were used. Table 1 shows the main results when using two, three or four features in terms of percentage of coincidences with each observer’s scores and with the median of the observers’ scores. Features $f_{1}$ and $f_{2}$ were used when testing the method with two features. After, we added $f_{3}$ and, finally, $f_{4}$. A high level of coincidence was achieved when using three and four features (in the latter case, the proposed method achieves the best results), whereas the level of coincidences for two features was acceptable.

**Figure 10:**
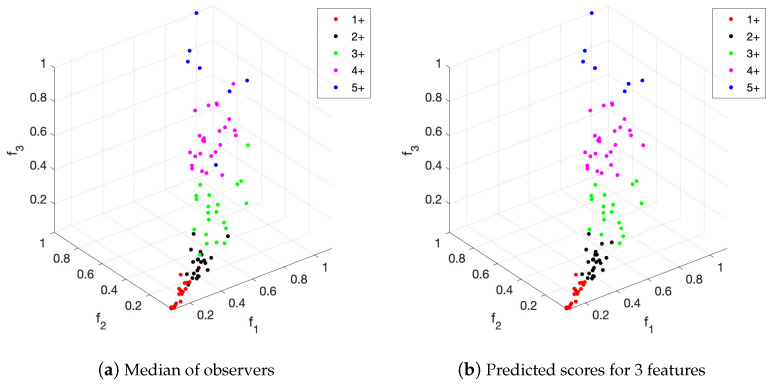
Scatter plot of the first three features together with (**a**) the median of the scores assigned by observers and (**b**) the predicted scores for these features after the clustering algorithm.

Figure 10 shows a graphical comparison of the predicted scores and the median of the observers’ scores versus the features values. For this, we considered the special case of when only the first three features are exploited, since a scatter plot in one figure is not possible for the case of four dimensions. Nevertheless, as shown in Table 1, the proposed method can also provide excellent results when only the first three features are exploited. Indeed, automated scoring achieves a high level of similarity with the reference truth, despite small differences.

**Figure 11:**
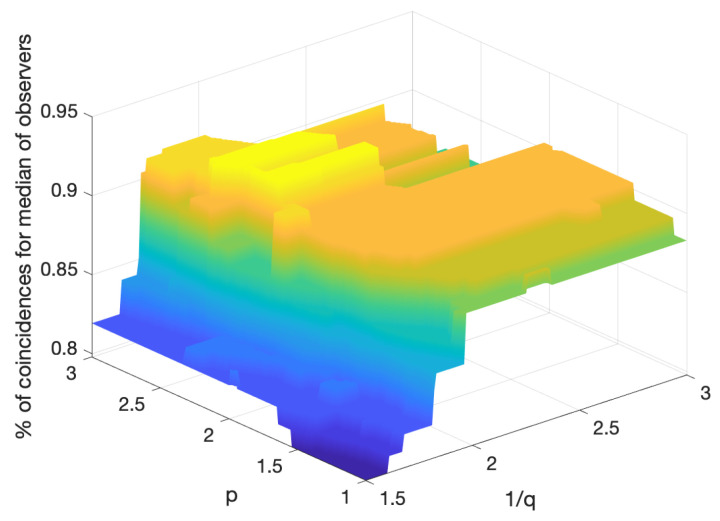
Percentage of coincidences of the scoring procedure with the median of the observers for the use of four features versus the parameters *p* (norm) and 1/q (root applied to the norm of the features vector for initialization).

A wide range of values are suitable for the parameters *p* (*p*-norm for initialization and distance in the clustering algorithm) and *q* (the power for reshaping the curve of the norm values of the feature vector). Figure 11 shows the coincidence percentage of the scores assigned by the proposed algorithm with the median of the scores assigned by the observers. For values of more than 90%, a low correlation between *p* and *q* can be observed. The parameter *p* can be selected from the interval $\left[1,3\right]$, which includes both the common Euclidean norm and 1-norm, while the parameter *q* is computed by means of the procedure described in Section 2.3. This leads us to conclude that the proposed method is quite robust in terms of the parameter selection, enabling an automated procedure for the scoring of IHC images.

Since feature $f_{2}$ is essential in our algorithm and involves the intensity matrix for the H stain, the proposed method is limited to biological samples that are stained with two stains. Thus, we cannot assure that the procedure achieves the best results when only exploiting $f_{1}$ and $f_{3}$ ($f_{4}$ would not make sense in this case) in the case where only a chromogen is used for staining.

## 4. Conclusions

In recent years, automated scoring methods have been developed for the evaluation of IHC images. However, these methods suffer from problems related to the need for researchers to intervene at a certain point in the process, the use of control or training images and the dependence of the results on the choice of certain parameters.

In the present work, we have proposed a completely unsupervised method that allows for scoring IHC images without the need for reference images and without depending on setting up several parameters. The procedure consists of a first stage of separating DAB and H stains. In this stage, PCA allows us to work in a lower dimensional space (a plane), which allows us to find the vectors associated with both stains in a simple and efficient way. Once these vectors and the intensity matrices of both stains are obtained, four characteristics are used to cluster the images, although it has been proven that the use of only three of them provides very good results; even the use of only two achieves acceptable results. The clustering method used (k-means) is based on automated initialization without the need for reference images or parameter setup, making it a robust, unsupervised method.

The proposed method has been tested on a database of 94 images, and the reference truth was taken from four expert observers. The scoring consisted of five categories, and the algorithm reached a mean correct prediction percentage of 87.23% from a maximum of 90.96% (due to the experts’ coincidence percentage, which limited the average maximum percentage of correct predictions). It would be straightforward to adapt the method to a different number of categories, as in our previous work.

As a future line of work, we think it would be interesting to find simple, fast and effective methods to locate nuclei and to develop new features related to DAB nuclei staining, since this could help to improve our results. On the other hand, for IHC images that only use DAB staining, the $f_{1}$ and $f_{3}$ features, together with newly defined features, should be tested.

## Figures and Tables

**Table 1:** Accuracy achieved by the proposed algorithm (% of coincidence with each observer’s scores). The use of two, three or four features was compared.

Reference		$f_{1}$, $f_{2}$ ^1^	$f_{1}$, $f_{2}$, $f_{3}$ ^2^	$f_{1}$, $f_{2}$, $f_{3}$, $f_{4}$ ^3^
Observer 1		86.17	89.36	90.43
Observer 2		78.72	79.79	78.72
Observer 3		85.11	88.30	89.36
Observer 4		86.17	89.36	90.43
	Mean of results ^4^	84.04	86.70	87.23
Observer’s median ^5^		88.30	91.49	92.55

^1^ p=1.5,q=1/1.8, one iteration. ^2^ p=1.9,q=1/1.75, two iterations. ^3^ p=2.5,q=1/2, three iterations. ^4^ Mean of above results. ^5^ Comparison with the median of the observers’ scores.

## Data Availability

The data that support this study are available from R.V.D. upon reasonable request.

## Abbreviations

The following abbreviations are used in this manuscript:

IHC	Immunohistochemistry
RGB	Red Green Blue
DAB	3,3′-Diaminobenzidine
H	Hematoxylin
CD	Color Deconvolution
NMF	Non-Negative Matrix Factorization
OD	Optical Density
PCA	Principal Component Analysis
